# Muriate of Ammonia in Hemicrania

**Published:** 1843-03

**Authors:** 


*Muriate of Ammonia in Hemicrania.*—Dr. Watson, in his clinical lectures, thus speaks of the Muriate of Ammonia in Hemicrania:

It is well worth knowing that muriate of ammonia is most serviceable in this form of hemicrania. Of the remedial properties of sal ammonia very little is known, at least very little was so until lately; its efficacy and the mode of administering it were first made known to me by an old apothecary of this city, who had, in innumerable cases, found it a sovereign cure. It should be administered in doses of half a drachm, or a scruple, and you will find that where persons complain of pain in the jaw and the whole side of the head, the pain freely yields to this dose of muriate of ammonia. I may add that in Germany this medicine is used in many cases where we use mercury, and for the same purposes, as in hepatic affections, and that it produces the required results without any of the inconveniences attending the use of mercury.

*Med. Ex.*, from *Prov. Med. Journ.*

